# Adaptive capacity in the implementation of disaster response village programme in Indonesia: Literature review

**DOI:** 10.4102/jamba.v15i1.1470

**Published:** 2023-09-18

**Authors:** Simon S. Hutagalung

**Affiliations:** 1 Department of Public Administration, Faculty of Social and Political Science, Universitas Lampung, Bandar Lampung, Indonesia

**Keywords:** adaptive capacity, community resilience, disaster response, disaster management

## Abstract

The disaster-resilient village programme, which aims to develop settlements with the autonomy to adapt and deal with disasters aims to promote disaster awareness. Several questions arise regarding the contribution of the programme to the development of the adaptive capacity of village communities, including: what types of adaptive capacity emerge as a result of the programme’s implementation, and what factors promote and inhibit the development of adaptive capacity? This study employs a comprehensive literature review. The initial step is to do a keyword search using Publish or Perish 8.2.3944.8118. This study includes a range of search phrases, including the phrase ‘disaster-resilience’ and keywords: community resiliency, disaster preparedness and disaster policy. The type of adaptive capacity that emerges from the implementation of the disaster response village programme incorporates the topic of flexibility predominantly, showing that this programme raises a diversity of adaption practices and possibilities in the community. This approach is influenced by the disaster-prone geographical characteristics of Indonesia. Practical gaps exist in the form of evaluating values in the development of similar disaster programmes, while theoretical gaps exist in the form of conceptual identification of cultural characteristics that may occur as a result of efforts to build adaptive ability through the programme. This article examines societal values that are affected by programmes.

**Contribution:**

This manuscript aims to add to the variety of disaster programme design initiatives requiring community resilience and sustainability. This sociocultural and disaster-related field is pertinent to the scope of this publication.

## Introduction

Impacts from natural hazards, human-caused disasters, climate change and social disputes can all have far-reaching effects on a community’s social system. This change calls for a transition away from a reactive catastrophe risk management strategy towards a more flexible, adaptive one. Effective communication, collaboration and coordination are essential in this paradigm for disaster management at the local, regional, national, multi-institutional and international levels (Medina 2016). This paradigm is consistent with an ecological framework based on values such as collaboration, social justice, empowerment and diversity respect (Gil-Rivas & Kilmer 2016). However, a study conducted by Tomio et al. (2014) shows that disaster preparedness is not yet adequate both at the household and community levels (Tomio et al. 2014). Therefore, the paradigm shift requires socio-political support from the community, especially those related to efforts to strengthen disaster adaptive capacity.

Himes-Cornell et al. (2018) found that areas rich in social, political and economic capital recover more quickly from disasters, allowing for a long-term transformation or recovery process. Utilisation of community social capital can support diverse efforts to enhance disaster preparedness (Himes-Cornell et al. 2018). According to the findings of additional research, the majority of community engagement techniques are effective in increasing preparedness to some degree. Practical and interactive approaches appear to be more effective than mass media campaigns for routine methods (Ryan et al. 2020). Communities must be educated on methods to support emergency preparedness, be adaptable and pay special attention to identified areas of vulnerability (Shannon 2015). These communities will require assistance such as emergency food supplies, emergency water plans, home medical supplies and evacuation plans (Stewart et al. 2017). This effort to strengthen the capacity of the disaster community will later be parallel with strengthening local disaster institutions and in line with the challenges of disaster response; the assignment of the appropriate task force to the appropriate location and time, so that the disaster response team must utilise adaptable local volunteers during the disaster response period (Hashemipour, Stuban & Dever 2017). As a means of influencing community preparedness for disaster risk, disaster management programmes must ensure the dissemination of sufficient and interactive information in the event of a disaster. This information should be easily accessible, comprehensive and tailored to the needs of the community (Abunyewah et al. 2020).

A community-based bottom-up approach to disaster risk management and the dissemination of disaster risk information that is suitably tailored to promote a proactive community-based resilience and disaster prevention framework is required to oversee this community capacity building (Aka et al. 2017). This programme is globally linked to community disaster management (CDM), encourages government, business and community members to work together to build resilience to prepare for and respond to catastrophes in Indonesia. In order to formalise this process, context-specific catastrophe groups have been set up to raise public awareness, disseminate information and keep natural and man-made disasters on the table (Ali et al. 2019).

Three mechanisms – social, functional and sequential – are employed in the community’s CDM-based approach to manage disasters. It is the social mechanisms that play the most significant role in catastrophe management, followed by the functional and sequential processes. Villagers get the organisational skills necessary to manage logistics, human resources and other coordinating tasks through their participation in a wide range of communal events (Pratama & Sariffuddin 2018). New tools and alternative funds for Disaster Risk Reduction have been developed thanks to public investment in community-based disaster risk financing in Indonesia. Disaster risk financing in Indonesia demonstrates that the village fund may support action plans to improve the community’s adaptive capacity and can also entice larger funding for mitigation initiatives in their environment (Srikandini, Prabandari & Rizal 2022). Although the amount is very small compared with the total budget received in the current year, village funds are relatively useful for strengthening adaptive capacity (Nur, Dirhamsyah & Fahlevi 2019). These CDM programmes include the Disaster Resilient Village Programme initiated by the Government of Indonesia.

One of the efforts made by the government of Indonesia to build disaster awareness is the disaster-resilient village programme, which is designed to build villages that have the independent ability to adapt and deal with disasters and to recover quickly from the impact of disasters if they are hit by a disaster (Antara News 2021). The existence of the disaster response programme should be able to build the capacity of the village community in dealing with disasters. However, there are some notes on the implementation of the program in several areas with several important points. The Disaster Resilient Village Programme is more dominantly implemented in a formative structural approach, where activities must be carried out in certain activities and durations so that in the future it needs to be encouraged to strengthen normative aspects originating from internal communities or community groups (Purwaningtyas 2021). This then creates an unsustainable pattern of volunteer regeneration and is able to become an agent of strengthening disaster awareness (Munir, Harsasto & Taufiq 2017). Many question that deserves to be examined regarding the contribution of the programme in building the adaptive capacity of village communities, there are several questions that then arise, namely: What are the forms of adaptive capacity that emerge from the implementation of the programme?; and What are the factors that are driving and inhibiting the development of adaptive capacity in the program implementing village communities? This question will be explored in more detail in this article.

## Literature review

Adaptive capacity has been the focus of several recent studies, and these studies have begun to add psycho-social and institutional components into their narrower analyses. This change acknowledges and aids in overcoming the shortcomings of prior methods of assessing adaptability. Yet there have been conversations that expand our understanding of the connection between adaptive capability and adaptation outcomes. The authors observe that the framework comprises six variables that explain how capability is translated and mobilised into action: risk attitudes, personal experience, authorities’ views and expectations, place-bound linkages, conflicting concerns and household composition and dynamics (Mortreux & Barnett 2017).

Long-term adaptation requires adaptive capacity to deal with climate change, disaster recovery and the possibility of future societal conflicts. In this case, the interdisciplinary perspective can be summed up as the interaction between the adaptive capacity of social systems and social ecology, which has not only the potential to introduce new methods and insights but also causes fragmentation, hinders methodological development and limits the transfer of insights into adaptation practices (Siders 2019). In simple terms, it can be understood that there is a relationship between socio-economic characteristics (which determine the various options available for adaptive capacity) and the negative impacts that occur as a result of natural hazards (Daramola et al. 2016). Another strategy involves combining community adaptive capacity with hazard, exposure and sensitivity considerations (Nguyen, Liou & Terry 2019). Several approaches were developed in line with post-disaster recovery efforts. Among them is an asset-based approach that enables communities to recover better after a disaster and adapt post-disaster. Communities can balance the goals of rehabilitation with the quality of support for adaptation to future changes. In fact, they are able to think of recovery before an event actually occurs through mitigation strategies (Freitag et al. 2014). This research shows that adaptive capacity has a role to strengthen community associations affected by disasters.

The identification of more minimal adaptive capacity can be established at the household level, for example in a household survey in rural China conducted by Xu et al. (2022). To determine and assess household adaptive capacity (HAC), an indicator-based methodology was implemented. The results of this study demonstrate the detrimental effects of catastrophe relocation on HAC. Research shows that despite resettlement’s seeming centrality in Chinese development efforts, it actually decreases rather than increases households’ ability to adapt to natural calamities (Xu et al. 2022). Others propose a more nuanced method of constructing adaptive capacity, breaking it down into five distinct areas: assets that people can draw on when necessary; strategic flexibility; the capacity to organise and act collectively; the capability to recognise and respond to change and the ability of organisations to decide whether or not to change (Cinner et al. 2018). For more detail, this categorisation can be observed in Table 1.

**TABLE 1:** Adaptive capacity categorisation and definition.

No	Categories	Definition
1	Agency	The ability and freedom to use these elements of adaptive capacity to actively determine their future is essential for successful adaptation to environmental change. When we talk about people’s ability to respond to environmental change on their own terms or as a group, we’re talking about agency, the fifth dimension of adaptive capacity.
2	Assets	Resources such as money, technology and services (like medical care) are examples of assets. Assets can either be privately held or publicly owned. When people have resources they can rely on, they are better able to adjust to change.
3	Organisation	In terms of adaptive capability, the study of social organisation captures the myriad ways in which societies are structured to foster communication, collaboration and the free exchange of ideas. People’s ability to adapt to change can be enhanced by the social support and access to information and resources provided through formal and informal partnerships between individuals, communities and organisations.
4	Flexibility	The adaptability domain of flexibility captures the variety of possible adaptation alternatives and indicates the capability for switching between adaption techniques. Adaptation to climate change is easier for more malleable organisations and people.
5	Learning	People’s learning represents their ability to create, assimilate and use new knowledge regarding climate change, adaptation pathways and strategies for coping with and adapting to uncertainty. Learning can take place inside and across many organisational, spatial and temporal scales, and it can be either experimental or experiential.

*Source*: According to Cinner, J.E., Adger, W.N., Allison, E.H., Barnes, M.L., Brown, K., Cohen, P.J. et al., 2018, ‘Building adaptive capacity to climate change in tropical coastal communities’, *Nature Climate Change* 8(2), 117–123. https://doi.org/10.1038/s41558-017-0065-x

Meanwhile, the following instruments and methods may be effective in bolstering adaptive capacity and disaster resilience; some references discuss whether this is the case:

- Evaluate and assess the strength of: underlining the significance of indicators of governance, risk assessment, knowledge and education, risk management and vulnerability reduction and disaster preparedness and response and highlighting the importance of exposure, sensitivity and adaptive capacity (Arbon 2014).
- Strengthening disaster resilience requires a multi-level, multi-stakeholder approach to risk governance, which can be achieved when donors and governments adopt such an approach. It has been shown that linking treatments that take place at multiple dimensions and levels is crucial (Djalante 2012).
- Community disaster resilience has been shown to be most effective when practitioners include gender in programming, tailor interventions to local conditions and guarantee meaningful participation from those most at risk (Alaerts 2020). Understanding the benefits and drawbacks for marginalised groups is crucial for achieving inclusive and participatory environments.
- Situational flexibility: Various crises present varying difficulties and openings. Good governance, gender equality and involvement with different social groups, conflict resolution, livelihood diversification and access to public infrastructure and services are all factors that have been shown to increase resilience (Janssen & Van Der Voort 2020).
- Insurance, loans, special funds, remittances and multi-year assistance are just some of the flexible funding tools that can help build financial resilience before, during and after a disaster (Combaz 2014).

The adaptive capacity of the system may be weakened by problems such as those highlighted in these descriptions, which include a lack of adequate infrastructure, complex interactions between institutions, reliance on external funding and inadequate data on human and material losses. So, we need to work harder to build adaptable competence and adaptive governance (Bakkour et al. 2015). Among them are efforts to develop the adaptive capacity wheel of resilience through public–private collaboration for disaster preparedness (Nguyen, Esteban & Motoharu 2021).

## Research methods and design

**FIGURE 1:**
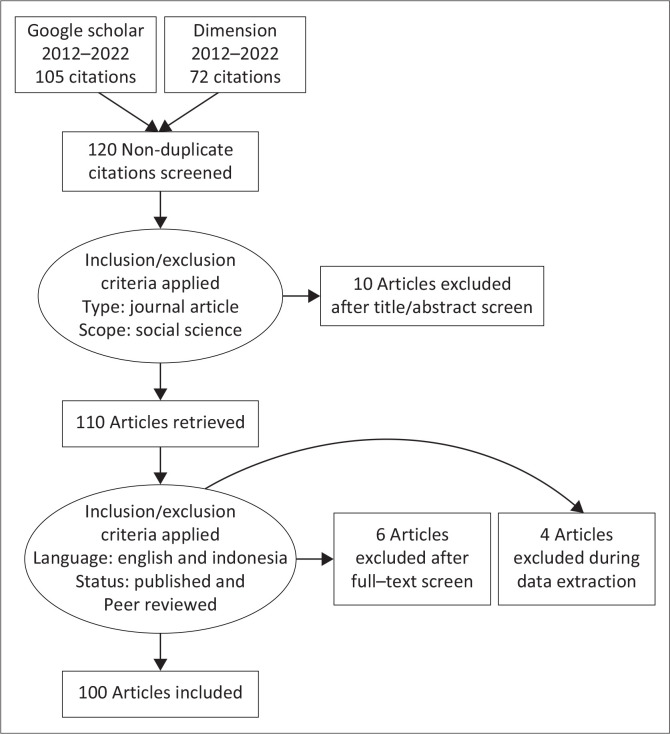
Prisma framework of adaptive capacity on village resilience programme.

This research applies a systematic literature review. The initial process begins with a keyword search using *Publish or Perish 8.2.3944.8118.* There are several search terms used in this study, including the use of title words: resilient to disasters, the use of keywords: village tough; disaster resilience; policy. Years of coverage (Years): all and other options (Other options) by issuing citations (exclude citations) and exclude patents (exclude patents). There are two database sources used: Google Scholar and Dimension. In the Google Scholar database, the scope of the publication year is 2012–2022 with a citation year of 10 years (2012–2022). The search results were 112 articles with 104 citations. Meanwhile, in the dimension database, the publication year ranged from 2012 to 2022, and the citation years were also 10 years (2012–2022). The search results were 72 articles with a total of 84 citations. The results of this keyword tracking were then followed up with the application of the Prisma Framework to filter out articles that were deemed irrelevant, ineligible and could not be analysed. Figure 1 is an application of the prism framework to the topic.

In the next process, the data for the 100 articles that want to be reviewed is stored in comma separated values (CSV) format and then displayed through the Microsoft Excel application and tidied up based on the article title, the author, year of publication, abstract and name of the journal that published the article, other information deemed irrelevant will be omitted. Furthermore, identification and analysis are carried out based on the categorisation framework of adaptive capacity in the village resilience programme and then grouped so that information can be obtained in the form of the distribution of quantities from each of these categories. Table 2 is the categorisation framework applied in this study.

**TABLE 2:** Adaptive capacity category and scope.

No.	Category	Scope
1	Agency	The ability and autonomy to organise in order to take control of their own destiny. Having the freedom to choose how you, as an individual or a group, react to environmental shifts
2	Asset	Assets are the resources that a person has access to, such as money, technology and services. Assets can either be privately held or publicly provided. When people have resources to fall back on, they are better able to adjust to change.
3	Organisation	The institutional frameworks that promote or stifle group dynamics, collaborative activity, and the exchange of information. The informal and formal links that bind people, groups and institutions Access to information and community resources
4	Flexibility	Switching between different adaptation strategies is possible, and the variety of available adaptations is accurately represented. Adapting to climate change requires both organisational and personal adaptability.
5	Learning	The ability to learn is a reflection of people’s ability to produce, assimilate and interpret new data concerning climate change, adaptation alternatives and strategies for coping with and managing uncertainty. Learning occurs inside and across different organisational, spatial and temporal scales, and it can be either experimental or experiential.

*Source*: According to Cinner, J.E., Adger, W.N., Allison, E.H., Barnes, M.L., Brown, K., Cohen, P.J. et al., 2018, ‘Building adaptive capacity to climate change in tropical coastal communities’, *Nature Climate Change* 8(2), 117–123. https://doi.org/10.1038/s41558-017-0065-x

No., number.

Meanwhile, in an effort to explain the Pushing and Inhibiting Factors in the Disaster Resilient Programme, open coding was carried out from articles that had been checked for feasibility and relevance. At the initial stage, direct categorisation of the scope of the concepts used is carried out, so that a distribution of answers is produced that can describe the diversity of quantities from each of the open categories. Furthermore, identification is carried out in a more patterned manner by referring to the five categorisations of adaptive capacity so that a conceptual mapping can be produced that shows the dominant and secondary scopes.

### Ethical considerations

This article followed all ethical standards for research without direct contact with human or animal subjects.

## Results and discussion

**FIGURE 2:**
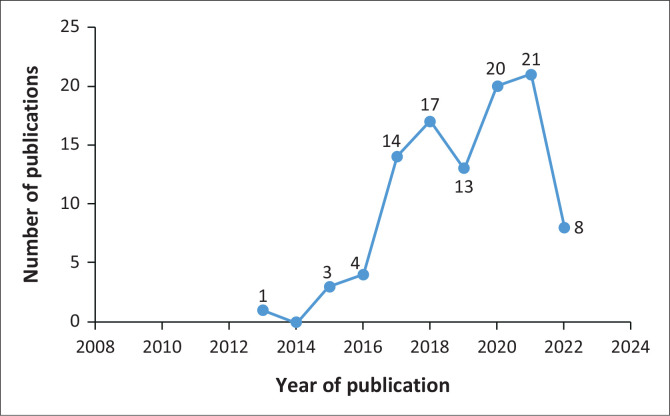
Distribution of publication document by years.

Based on the data obtained, it can be observed that the distribution of research within the scope of the topic of this disaster resilience programme have started from 2013 with 1 publication and then slowly increased from 2016 with 4 publications and then continues to increase until 2021 with the number of publications of 21 documents. Meanwhile, for 2022, it cannot be concluded because it is currently running. This distribution can be seen from Figure 2.

Publications conducted in that time span have various research orientations, from those that attempt to analyse the capacity of village institutions, identify disaster mitigation efforts and conduct model analysis in strengthening disaster-resilient village institutions. The emergence of these publications then gave rise to feedback trying to cite several documents that are considered relevant and qualify to be used as references. The following is presented in Table 3 as some of the publications identified as having a high citation quantity.

**TABLE 3:** Most cite articles on resilience village programme topic.

No	Cites	Authors	Year
1	11	A Buchari, MB Santoso, N Marlina	2017
2	11	W Budiarti, E Gravitiani, M Mujiyo	2017
3	8	M Munir, P Harsasto, A Taufiq	2017
4	7	A Najib, HK Rahmat	2021
5	6	YS Hijri, W Kurniawan, …	2020
6	6	G Saptadi, H Djamal	2012
7	5	SPM Yusuf	2015
8	4	H Habibullah	2013
9	4	S Utami, K Ekasari, RM Saputra	2020
10	3	B Prakoso, IDKK Widana, …	2021

The first rank publication containing the most articles (11 citations) investigates the capacity development of disaster-resilient village institutions in Garut district; the article reviews the independent ability to adapt and face disaster threats in several villages that are research locations (Achmad & Santoso 2018). The second most cited article examines flood mitigation efforts in the Samin sub-watershed through the development of disaster-resilient communities (Budiarti, Gravitiani & Mujiyo 2017). This publication seems to be widely used as a reference for writing other articles related to the topic of flood mitigation or the topic of disaster-resilient communities (11 citations). In the third position appears an article with the topic of Evaluation of the Implementation of the Disaster Resilient Village Program in Kendal Regency, this article seeks to identify and analyse the disaster resilient village programme, which is a programme from the government for community groups in the village (Munir et al. 2017). Other publications appear to have varied topics and scope of analysis, from evaluating disaster resilience programmes (Najib & Rahmat 2021) to more specific ones such as policy making in disaster resilient villages (Hijri, Kurniawan & Hilman 2020). Each document has its own appeal so that it is used as a reference for other published documents. However, what will be the focus of this article is the detail of the substance that can be associated with several categorisations of the concept of adaptive capacity.

### Categorisation of adaptive capacity in the disaster resilient programme

The identification and categorisation carried out show that the publications conducted with the topic of analysis on disaster resilience programmes have a dominant substance orientation, which is in the flexibility category (28%) followed by the agency category (25%) and organisation (24%).

**FIGURE 3:**
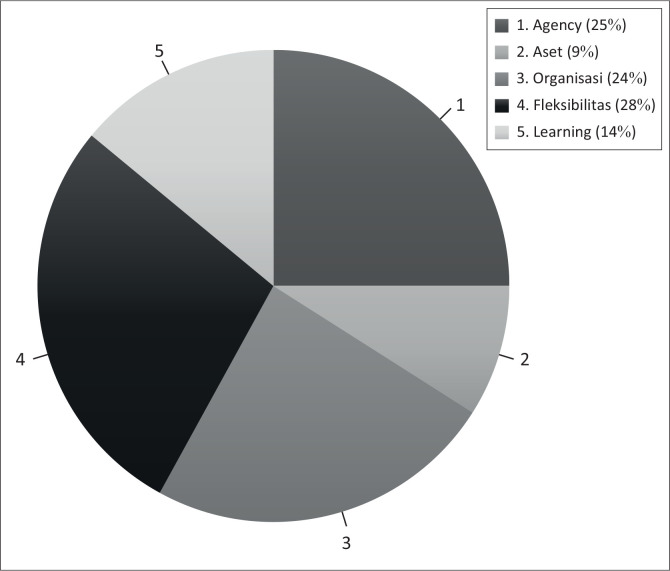
Distribution diagram of study categories covering adaptive capacity in disaster response programmes.

Articles that have a topic in the scope of flexibility seem to raise a discussion about adaptation strategies within a particular group that can create a unique form of choice of action. In addition, this topic is related to studies that try to explain the ability of a particular organisation or group to adapt more quickly to climate change or post-disaster. Then, in the article that has a theme in the agency category, it seems to review the strength and ability of a community to move to overcome the challenges and problems they face. The adaptive capacity in this article appears to be a form of their experience in overcoming challenges and obstacles in their social activities. In addition, it also includes a review or analysis of individual or collective abilities in managing their environment, including articles related to the study of leadership best practices or the example of certain individuals. The implication on adaptive capacity is the foundation for strengthening the resilience and adaptive governance that exists in society. The distribution of these two largest categories can be compared visually through Table 4 and Figure 3.

**TABLE 4:** Distribution of categories on adaptive capacity in resilience village programmes.

No	Category	Total	Percentage
1	Agency	25	25.00
2	Assets	9	9.00
3	Organisations	24	24.00
4	Flexibility	28	28.00
5	Learning	14	14.00

**Total**		**100**	**-**

The organisational category is in third place with 24% of the articles analysed. In this category, the articles written have a review of the practice of cooperation, organising and sharing experiences that occur in the community. In addition, reviews are related to formal and informal interactions between individuals, communities and other organisations in order to strengthen access to knowledge and resources. In this category, there are articles that review formal village organisations that are faced with the challenge of following the development of programme implementation. Furthermore, the category of learning or learning with a composition of 14%, which includes reviews or studies related to learning or education efforts from a group or community to adapt, manage and live together with the challenges of uncertainty facing disasters. The studies covered in this theme relate to experiential learning or testing efforts and also relate to facts or events in various organisational settings. This learning effort can be understood as one of the actions needed to build the adaptive capacity of individuals or communities.

In the asset category, only about 9% of all articles analysed. This category includes discussion of various assets such as financial, technology and human resources that can be accessed by individuals or community groups. Then includes a study on the topic of public and private asset ownership, where ownership of these assets can strengthen the capacity of individuals and communities to adapt in dealing with certain disasters or crisis conditions. Other studies included in this category include reviews of the carrying capacity of assets or potential assets owned by certain community groups or individuals in the post-disaster rehabilitation phase so that they are more optimal in dealing with change.

From the identification, several descriptions can be known as follows:

- The form of adaptive capacity that emerges from the implementation of the village disaster response programme is dominantly loaded with the topic of flexibility, this indicates that this programme raises various practices related to adaptation strategies and various adaptation options that arise in the community. This programme has an appeal to be reviewed or described in the aspect of community and individual changes in adapting to unexpected conditions.
- The lack of attention to the topic of assets in the implementation of disaster response programmes indicates that this programme is not yet dominant in creating attractiveness regarding the role or position of asset support for strengthening adaptive capacity. This is also an opportunity for similar research to evaluate disaster response programmes in Indonesia.

### Identification of supporting and inhibiting factors in the disaster resilient programme

Through the analysis, we can identify several supporting and inhibiting factors in the implementation of the disaster response programme. Efforts to identify these two forms of factors are expected to clarify the forms of obstacles that still interfere with strengthening the adaptive capacity of the programme implementing group. The identification can be observed in more detail in Table 5.

**TABLE 5:** Identification of supporting and inhibiting factors in programme.

No	Categorisation			
	Supporting	*N*	Inhibiting	*N*
1	Urgency of the programme for the community	30	Perceptions of fulfilling activities solely	27
2	The community’s need to be adaptive	22	The view that disasters cannot be managed	20
3	Anticipatory policies	17	Commitment to sustainability	16
4	Means for strengthening community survival	10	Commitment to education	14
5	Caring attitude and social solidarity	9	Lack of facilities	12
6	Worrying about material loss	7	Unsustainable funding support	7
7	Worrying about personal safety	5	Government care is minimal	4

**Total**		**100**		**100**

From Table 5, it can be observed that there is still a dominant perception among the programme target groups that programmes that have noble goals are still considered routine activities, where the sustainability process will depend solely on the fulfillment of administrative achievements. Then, the views of the target group and programme implementers are still dominant, who see that a disaster cannot be managed and humans can only surrender to pray when faced with a disaster. This view shows that the implementation of the programme has not intervened in the belief aspect or the target group’s belief in the disaster background and opportunities to minimise the risk. This obstacle will be related to efforts to strengthen the adaptive capacity of communities in a location.

Meanwhile, the urgency of the programme and the community’s need to be adaptive were the dominant supporting factors identified in the various articles. The view is related to the urgency of the programme if it is associated with the geographical condition of the country of Indonesia, which is prone to disasters, so that efforts to strengthen the capacity of the community are one of the efforts to minimise the adverse effects of the disaster. This is in line with the view that this programme has substance that reflects the community’s need to be more adaptive in dealing with potential disasters or threatening changes. These two dominant factors indicate that the initial perception of this programme is positive, based on the real needs of the community and very urgent to implement. However, the implementation process that is varied in each community causes some of these initial perceptions to turn negative and then become factors that hinder programme achievements.

## Conclusion

The form of adaptive capacity that emerges from the implementation of the disaster response village programme in Indonesia dominantly contains the topic of flexibility, indicating that this programme raises a variety of practices related to adaptation strategies and a variety of adaptation options that emerge in the community. In addition, the lack of attention to the topic of assets in the implementation of disaster response programmes indicates that this programme has not yet dominantly raised the appeal related to the role or position of asset support for strengthening adaptive capacity. The inhibiting factors in the implementation of disaster response programmes include the perception that develops in the programme target groups regarding the implementation of programmes that are considered routine activities. Otherwise, there is a target group and programme implementers perception that disasters cannot be managed, and humans can only surrender to pray when faced with disasters. It shows that the programme implementation has not intervened in the belief aspect of the target group on the background of disasters and opportunities to minimise the risks. Meanwhile, supporting factors for programme implementation include the urgency of the programme and the need for the community to be adaptive, this view is related to the geographical conditions of Indonesia, which are prone to disasters.
